# Inverse Identification of Single-Crystal Plasticity Parameters of HCP Zinc from Nanoindentation Curves and Residual Topographies

**DOI:** 10.3390/nano12030300

**Published:** 2022-01-18

**Authors:** Pham T. N. Nguyen, Fazilay Abbès, Jean-Sébastien Lecomte, Christophe Schuman, Boussad Abbès

**Affiliations:** 1 The University of Danang, University of Science and Technology, Da Nang 550000, Vietnam; nptnhan@dut.udn.vn; 2 MATériaux et Ingénierie Mécanique (MATIM), Université de Reims Champagne Ardenne, 51100 Reims, France; fazilay.abbes@univ-reims.fr; 3 Laboratoire d’Étude des Microstructures et de Mécanique des Matériaux (LEM3), CNRS UMR 7239, Université de Lorraine, 57000 Metz, France; jean-sebastien.lecomte@univ-lorraine.fr (J.-S.L.); christophe.schuman@univ-lorraine.fr (C.S.); 4 Laboratory of Excellence on Design of Alloy Metals for low-mAss Structures (DAMAS), Université de Lorraine, 57000 Metz, France

**Keywords:** crystal plasticity, CPFEM, nanoindentation, parameter identification, zinc

## Abstract

This paper investigates the orientation-dependent characteristics of pure zinc under localized loading using nanoindentation experiments and crystal plasticity finite element (CPFEM) simulations. Nanoindentation experiments on different grain orientations exhibited distinct load–depth responses. Atomic force microscopy revealed two-fold unsymmetrical material pile-up patterns. Obtaining crystal plasticity model parameters usually requires time-consuming micromechanical tests. Inverse analysis using experimental and simulated loading–unloading nanoindentation curves of individual grains is commonly used, however the solution to the inverse identification problem is not necessarily unique. In this study, an approach is presented allowing the identification of CPFEM constitutive parameters from nanoindentation curves and residual topographies. The proposed approach combines the response surface methodology together with a genetic algorithm to determine an optimal set of parameters. The CPFEM simulations corroborate with measured nanoindentation curves and residual profiles and reveal the evolution of deformation activity underneath the indenter.

## 1. Introduction

Zinc metal has several characteristics that make it a well-suited corrosion protective coating for iron and steel products [1,2]. Its high corrosion resistance in different environment conditions accounts for its successful use as a protective coating on a variety of products and in many exposure conditions, especially in automotive and building applications [3,4,5]. In the automotive industry, hot-dip galvanized sheets, with 100% of zinc, or GALFAN (with 95% Zinc and 5% Al) are the most used products [6,7].

Zinc crystals have a hexagonal close-packed structure (HCP). The unit cell of the HCP lattice is a hexagonal prism which has two hexagonal bases with sides of length a and height c. The HCP unit cell can be imagined as a hexagonal prism with an atom on each vertex, and three atoms in the center. Compared to other HCP metals such as titanium or magnesium, zinc is characterized by a high c/a lattice ratio (Zn: c/a=1.856, Mg:c/a=1.624, Ti:c/a=1.588) and exhibits a strong anisotropic behavior. For zinc, the plastic deformation modes are basal, prismatic, and pyramidal slip systems and compression twinning. The basal glide $B\langle a\rangle \;\left\{0001\right\}\langle 11\bar{2}0\rangle$ is the easiest deformation mode, followed by the pyramidal $\pi 2\langle c+a\rangle \;\left\{1\bar{1}22\right\}\langle 11\bar{2}\bar{3}\rangle$ slip system [8,9,10,11,12].

To model the anisotropic mechanical behavior of zinc, it is necessary to know both the texture and deformation mechanisms [13,14]. Critical resolved shear stresses (CRSSs) represent the deformation mechanisms: slip begins when the shear stress on a slip system reaches its critical value CRSS. The most direct way to establish the CRSS is to conduct a macroscopic tension or compression test on a suitably oriented single-crystal sample. A considerable body of work has been undertaken, predominately in the 1950s, 1960s, and 1970s, to establish the fundamentals of CRSS values for the important slip systems in the pure HCP metals. Another method is the combination of deformation experiments of polycrystalline samples along with crystal plasticity simulations using crystal plasticity finite element models. The CRSSs of the various slip systems are adjusted in the model using an iterative scheme, so that a good fit is obtained to the stress–strain response from macro-scale experiments [15,16] or the load-displacement data obtained by deforming small volumes with nanoindentation tests [17,18].

The CRSS of zinc crystal has been investigated by many authors, showing a wide range value for all orientations in the standard stereographic triangle. Moreover, the CRSS significantly depends on the contents of alloying elements [19] and the testing temperature, as well as on the thermal treatment [20]. In 1949, Harper and Cottrell [21] studied the effects of various surface treatments on the plastic properties of zinc crystals. The CRSS value of the basal slip system was about 0.33 MPa for electrolytically polished specimens and up to 0.64 MPa for oxidized specimens. Edwards and Washburn [22] analyzed the relative amount of hardening on active and latent systems of the basal plane of zinc crystals. The obtained value of CRSS of the basal slip system was 0.28 MPa. Bell and Cahn [8] examined the dynamics of twinning and the interrelation of slip and twinning in zinc crystals. Through the tension tests on zinc samples, they were the first to identify the pyramidal π2 slip, for which the critical resolved shear stress was 10 to 15 MPa. Adams et al. [23] carried out compression tests to observe and measure the strain, dislocation mobility, and dislocation density on basal and second-order pyramidal systems in 99.999% zinc single crystals at room temperature. Slip bands are formed at stresses greater than 0.05 MPa in the basal system, and 6 MPa in the second-order pyramidal system. Etch pit observations indicated that the average dislocation density increases linearly with strain [23]. Rogers and Roberts [24] analyzed the plastic anisotropy of zinc sheets in crystallographic terms. They realized that the variation of yield stress with the direction of tensile stress follows the Schmid relationship for the systems sustaining maximum resolved shear stress. With uniaxial tension, deformation occurred mainly by basal plane slip and twinning, with the importance of the latter mode increasing as the angle of the tensile axis to the rolling direction approaches 90°. Then, deformation twinning accompanied by complete change in the preferred orientation is the main feature. Barendreght and Sharpe [13] carried out an experimental investigation on pure zinc specimens to test the hypothesis that the CRSS was independent of the resolved normal stress on the active slip system plane. The CRSS was found to be about 0.7 MPa for the basal slip system. The resolved normal stress was varied independently for crystals of constant orientation by applying a uniform biaxial tension stress through slitted rubber strips glued to a flat tension sample. They found that the CRSS is constant until the resolved normal stress on the active slip system becomes twice the resolved shear stress, and then, for a further increase in the resolved normal stress, the critical resolved shear stress begins to decrease. Philippe et al. [10] described the texture and microstructure evolution during cold rolling of zinc alloys (Zn-0.16% Cu-0.076%Ti) relying on the Transmission Electron Microscopy (TEM) tool and the Taylor model. For this alloy, the best fitting was obtained with the following ratios: $\tau_{prism}/\tau_{basal}=15$, $\tau_{py\langle c+a\rangle }/\tau_{basal}=10$, and $\tau_{twin}/\tau_{basal}=30$. Diot et al. [25] analyzed the texture gradient in Zn-0.165%Cu-0.07%Ti cold-rolled sheets. They observed that in the rolling process, the texture gradient is probably due to the shear stress and the friction at the surface. In a similar way, Philippe et al. [10] defined the ratios of the critical shear stresses. In 2004, Zhang et al. [26] observed the development of the crystallographic textures in a Zn-0.17%Cu-0.08%Ti alloy sheet during asymmetric rolling. The ratios of CRSSs used for their simulations by a viscoplastic Taylor model were as follows: $\tau_{prism}/\tau_{basal}=15$, $\tau_{py\langle c+a\rangle }/\tau_{basal}=2$, and $\tau_{twin}/\tau_{basal}=30$. They saw that asymmetric rolling in the final pass should improve the mechanical characteristics of the ZnCuTi sheet, notably its bendability in view of industrial applications.

Parisot et al. [27] used a 3D finite element crystal plasticity model (CPFEM) to analyze deformation mechanisms of a thin zinc coating on a hot-dip galvanized steel sheet. The CRSSs used for finite element simulations were: $\tau_{basal}=1.5\;\mathrm{MPa},$ $\tau_{py\langle c+a\rangle }=10\;\mathrm{MPa}$, $\tau_{prism}=22.5\;\mathrm{MPa},$ and $\tau_{twin}=25\;\mathrm{MPa}$. Parisot et al. [28] compared the plastic slip and twinning activity in the zinc grains of an untempered cold-rolled coating, a tempered cold-rolled coating, and a recrystallized coating with the response of the corresponding bulk low-alloyed zinc material. The CRSSs used were for the basal slip system family with the lowest initial CRSS ($\tau_{0}=1.5\;\mathrm{MPa}$), for pyramidal π2 ($\tau_{0}=15\;\mathrm{MPa}$), for pyramidal π1 ($\tau_{0}=20\;\mathrm{MPa}$), for prismatic slip ($\tau_{0}=22\;\mathrm{MPa}$), and for twinning ($\tau_{0}=25\;\mathrm{MPa}$). The results showed that the deformation of zinc coatings on hot-dip galvanized steel sheets proceeds through the activation of many slip systems belonging to several slip system families. Basal slip was not the main deformation mechanism, except in the recrystallized coating. Pyramidal π2 slip was the major deformation mode in both coatings. In contrast, the deformation of the reference bulk zinc alloy mainly involved basal slip, as expected from the literature on single and polycrystalline pure or alloyed zinc [27,28].

Boczkal and Mikułowski [20] investigated the precipitation hardening of Zn + 0.1%Ti single crystals deformed on the basal system $\left(0001\right)\langle 11\bar{2}0\rangle$, including the mechanical properties (critical resolved shear stress and work-hardening coefficient) and a thermodynamic parameter (the activation volume). Based on compression tests, the investigation showed a great dependence of the value of CRSS and the work-hardening coefficient on the temperature for the $\left(0001\right)\langle 11\bar{2}0\rangle$ slip system: CRSS was from 12.5 to 5 MPa when the testing temperature changed from 77 to 473 K, as well as on the thermal treatment: CRSS changes in the range of 7.3 to 12.2 MPa. In 2005, Vincent et al. [29] studied the development of crystallographic textures in a galvanized zinc sheet during asymmetrical skin-pass. The material composition of the zinc layer was Zn + 0.2%Al. To obtain good simulation results, the authors used the ratios of the CRSS on slip and twin systems as follows: $\tau_{prism}/\tau_{basal}=15$, $\tau_{py\langle a\rangle }/\tau_{basal}=10$, $\tau_{py\langle c+a\rangle }/\tau_{basal}=10$, and $\tau_{twin}/\tau_{basal}=8$.

Borodachenkova et al. [30] carried out modeling and experimental studies on the mechanical behavior and texture evolution of a Zn alloy (Zn + 0.08%Cu + 0.1%Ti) subjected to deformation involving reverse loading at room temperature. A viscoplastic-self-consistent (VPSC) polycrystal model was applied for the simulation of multiple slip and twinning modes in the monotonic shear test and shear-reserve test. The identified hardening parameters were reasonable, in line with previous studies [26,28]. The estimated CRSSs on basal, prismatic, and pyramidal slip systems and twinning were: 7, 180, 75, and 200 MPa, respectively. Hagihara et al. [11] studied the deformation behavior of Zn single crystals, in which the formation of deformation kink bands occurred owing to compression tests using the crystal plasticity finite element method proposed by Peirce et al. [31]. The CRSS for the pyramidal π2 used in their simulations was 20 MPa.

Recently, Cauvin et al. [12] used an inverse analysis to identify the critical resolved shear stress as well as the micro-scale crystal parameters for the Zn-0.1–0.2%Cu-Ti alloy based on uniaxial tensile tests. The uniaxial tensile tests were carried out for different zinc sheets cut out along various orientations of the rolling direction (0°, 45°, and 90°). The Covariance Matrix Adaptation-Evolution Strategy (CMA-ES) genetic algorithm was used for the identification process by comparing the simulated and experimental results in terms of obtained tensile curves along the three rolling directions. The deformation modes considered in their model were: B〈a〉, π2〈c+a〉, and tensile twinning $\left(10\bar{1}2\right)\langle \bar{1}011\rangle$. The CRSSs obtained were, respectively: 1, 47.94, and 106.25 MPa.

The critical resolved shear stress values of the relative activity of different modes issued from the literature are listed in Table 1. It was found that there is a wide range of CRSS values in previous studies. This means that the exact deformation mechanisms in zinc metal, dislocation activity on specific slip systems, and twinning activation have not yet been completely understood.

Nanoindentation is a technique which can be used to measure the mechanical properties of materials with high precision. Also called instrumented indentation tests (IIT), it consists in continuously measuring and subsequently analyzing an applied load, *P*, and the corresponding penetration depth, *h*. The test procedure can either be force-controlled or displacement-controlled. With this technique, it is possible to estimate the elastic properties of the material being indented, in addition to hardness, *H*, defined as *P* divided by the projected area of the impression created by the indenter. Nanoindentation tests can be used for a wide range of applications, such as: study of mechanical properties of cement-based materials [37,38,39], study of surface coatings and thin films [40,41,42], study of Micro/Nano-Electro-Mechanical Systems (MEMS and NEMS) [43,44,45], and single-crystal plasticity analysis [46,47,48]. The most popular method for the analysis of raw data was proposed by Oliver and Pharr [49]. To determine elastic–plastic material properties, inverse analysis based on FEM simulations and measurements of nanoindentation responses is widely used [50,51]. The dimensionless analysis is also a popular method used to extract the stress–strain relationships from the load-displacement responses measured from indentations and nanoindentations [52,53,54].

In previous works [55,56], we have developed a crystal plasticity model to determine the critical resolved shear stresses and hardening parameters for the observed deformation mechanisms in pure zinc based on the solution of an inverse problem, in which we coupled 3D numerical simulations of the nanoindentation test to genetic algorithms to solve an optimization problem using load-penetration depth curves. As a natural extension of these previous works, we propose a new parameter identification methodology by incorporating the penetration depth profiles in the optimization process, which is a richer observation than the indentation curve. We have also leveraged an expensive function optimization strategy of constructing a surrogate model. By searching a response surface using the MOGA-II genetic algorithm (GA) and selectively updating the response surface using the CPFEM, a robust set of parameters is identified from nanoindentation curves and residual topographies with limited CPFEM calls.

The remainder of this paper is organized as follows: In Section 2, we present the material and the experimental conditions, in Section 3 we detail the CPFEM model and the identification methodology, and the main results and the discussion are presented in Section 4 and Section 5, respectively. Finally, we conclude with a summary of the findings.

## 2. Experiments

The material used in this work is a high-purity (99.99%) polycrystalline zinc, provided by Goodfellow (Lille, France). Made by cold rolling without recrystallization annealing, it is in the form of sheets of one millimeter of thickness. The rolling direction (RD) is marked on the sheet in indelible ink. This initial state was annealed in the laboratory before use. This material is called the as-received annealed cold-rolled sheet, named “AR”.

The chemical composition of the as-received plates is provided in Table 2 (the values are in parts per million (ppm), Goodfellow’s analysis of the batch either on the final material or during manufacture). It is shown that the composition of addition elements is very low and, consequently, will be assumed to be non-influential on the mechanical properties of zinc.

Microstructure characterization was performed with a field emission gun Jeol-6500F scanning electron microscope (SEM), equipped with an Electron Back Scattered Diffraction (EBSD) detector (JEOL Europe SAS, Croissy-sur-Seine, France) and the Aztec HKL software package (Aztec, Oxford Instruments, Abingdon, Oxfordshire, UK) for data acquisition. Samples for EBSD examination were first ground with sandpaper, and then were polished with 1 μm diamond paste to a mirror finish. To achieve the surface quality required for nanoindentation tests and EBSD examination (i.e., deformation-free, and scratch-free surface), a chemo-mechanical polish with colloidal silica suspension (~40 nm) on a porous neoprene polishing cloth for about 35 nm was used. An atomic force microscope (AFM) Nanos ScanPanel (Bruker Nano GmbH, Berlin, Germany) was used in contact mode to measure the roughness of the polished surface and to visualize the nanoindentation imprints.

**Figure 1:**
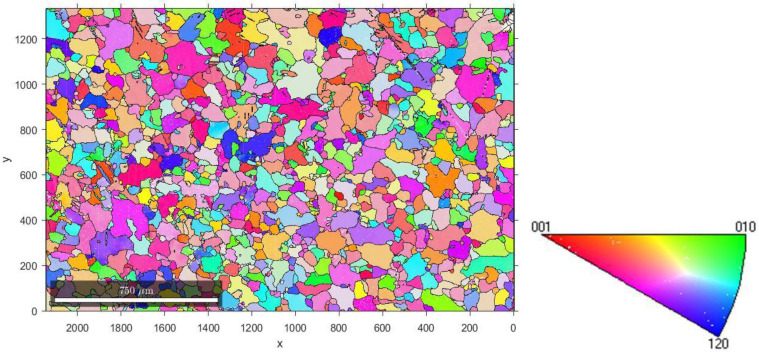
EBSD mapping of the selected area for the AR specimen and associated standard stereographic triangle.

Figure 1 illustrates the EBSD mapping obtained on a sample taken from the central zone of the zinc sheet in the as-received state (AR).

Nanoindentation tests were performed using a 90° cono-spherical diamond indenter (Young’s modulus E = 1141 GPa, Poisson’s ratio ν = 0.07) with tip radius of 1 μm, mounted in an NHT-2 nanoindenter (CSM Instruments, Peseux, Switzerland). Indentations were carried out in load-control mode. The indentation strain rate in the loading stage of this study was controlled to be 0.05 s^−1^. In practice, the applied load, *P*, on the indenter is accurately controlled, while the penetration depth, *h*, is simultaneously measured by recording the indenter’s displacement into the grain surface. To avoid grain boundary influence, all the indents were in the mid-zone of individual grains. Grain boundary interaction effects are thus neglected, and the use of the single-crystal plasticity framework is justified.

**Figure 2:**
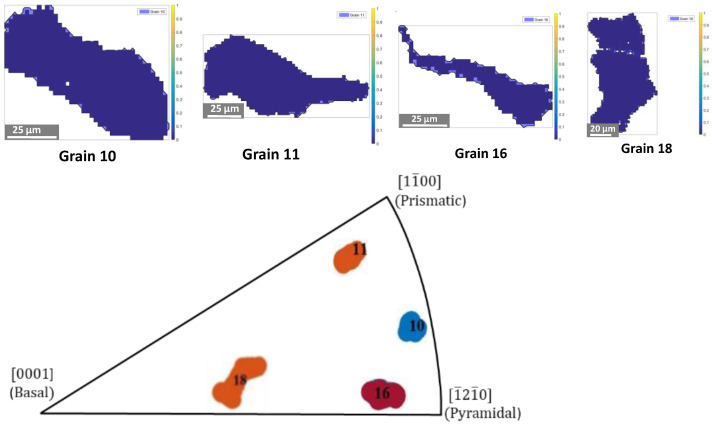
Shape and location in the standard stereographic triangle of the selected grains.

We carried out nanoindentation tests on different grains numbered from #1 to #18. We selected grains #10, #11, #16, and #18, corresponding to different crystallographic orientations provided in Table 3. The shape extracted from the EBSD map and the location in the standard stereographic triangle of the selected grains are shown in Figure 2. The grains for which the <c> axis (i.e., <0001>) is parallel to the normal direction of the sheet are called basal grains. Grains for which the $\langle \bar{1}2\bar{1}0\rangle$ direction is parallel to the normal direction of the sheet are called pyramidal grains. Finally, grains for which the $\langle 01\bar{1}0\rangle$ direction is along the normal direction of the sheet are called prismatic grains.

## 3. Modeling

### 3.1. Crystal Plasticity Constitutive Equations

An anisotropic stiffness tensor, ℂ, is considered to model the zinc elastic response. It is described by five components ($C_{11}$, $C_{33}$, $C_{44}$, $C_{12}$, $C_{13}$), as presented in Table 4 and obtained from the data compiled by Tromans [57]. In the absence of twinning, the total deformation gradient (F) can be decomposed to the elastic ($F^{e}$) and plastic ($F^{p}$) parts:

(1) $$F=F^{e}F^{p}$$

The plastic shear strain is assumed to occur only on each of the 12 HCP slip systems (3 basal systems $\left\{0001\right\}\langle 11\bar{2}0\rangle$, 3 prismatic systems $\left\{10\bar{1}0\right\}\langle 11\bar{2}0\rangle$, and 6 pyramidal $\pi_{2}$ systems $\left\{11\bar{2}2\right\}\langle 11\bar{2}3\rangle$), characterized by the slip directions, $s^{\alpha }$, and the normal to the slip planes, $n^{\alpha }$:

(2) $${\dot{F}}^{p}F^{p}{}^{-1}=\overset{12}{\underset{\alpha =1}{\sum }}{\dot{\gamma }}^{\alpha }s^{\alpha }⊗n^{\alpha }$$

The shear rate, ${\dot{\gamma }}^{\alpha }$, on slip system *α* is chosen as a Norton power law [58]:

(3) $${\dot{\gamma }}^{\alpha }={\dot{\gamma }}_{0}^{\alpha }{\left|\frac{\tau^{\alpha }}{\tau_{c}^{\alpha }}\right|}^{n}\mathrm{sign}\left(\tau^{\alpha }\right)$$

where ${\dot{\gamma }}_{0}^{\alpha }$ is a reference shear strain rate, n represents the sensitivity of the material to the strain rate, and $\tau_{c}^{\alpha }$ is the CRSS of the slip system *α*.

The dislocations slipping initiation is governed by the resolved shear stress, $\tau^{\alpha }$, defined as:

(4) $$\tau^{\alpha }=\sigma :m^{\alpha },\;\mathrm{with}\;\;m^{\alpha }=\frac{1}{2}\left(s^{\alpha }⊗n^{\alpha }+n^{\alpha }⊗s^{\alpha }\right)$$

where σ is the Cauchy stress tensor and $m^{\alpha }$ is the Schmid tensor.

The constitutive description is complemented with the rate of evolution of CRSS, ${\dot{\tau }}_{c}^{\alpha }$, which is assumed to have a saturation-type hardening form [59]:

(5) $${\dot{\tau }}_{c}^{\alpha }=h_{0}^{\alpha }\left(1-\frac{\tau_{c}^{\alpha }-\tau_{0}^{\alpha }}{\tau_{s}^{\alpha }-\tau_{0}^{\alpha }}\right)\dot{\gamma }$$

where $\dot{\gamma }=\underset{\alpha }{\sum }\left|{\dot{\gamma }}^{\alpha }\right|$ is a net shear strain rate within the crystal, and $h_{0}^{\alpha }$, $\tau_{0}^{\alpha }$, and $\tau_{s}^{\alpha }$ are slip system hardening parameters which are taken to be identical for all slip systems.

### 3.2. Crystal Plasticity Finite Element Simulation of the Nanoindentation Experiment

Three-dimensional finite element simulations of the nanoindentation experiments were performed using the commercial software ABAQUS. The zinc grain has been modelled as a cylinder of 20 μm diameter and 20 μm height. To avoid grain-boundary effects, the bottom and surrounding surfaces of the specimen were constrained. The cono-spherical indenter was modelled as a fully rigid body, with a tip radius of 1 μm and a half apex angle of 45°. The indenter was only allowed to move in the indentation direction. A friction coefficient of 0.1 was assumed between the indenter and the substrate. The same value was used by Long et al. [53] and Zhao et al. [60]. Even if friction is known to play a minor role in the load vs. displacement response prediction [61], the use of a friction coefficient enhances convergence by limiting mesh distortion in the contact area.

**Figure 3:**
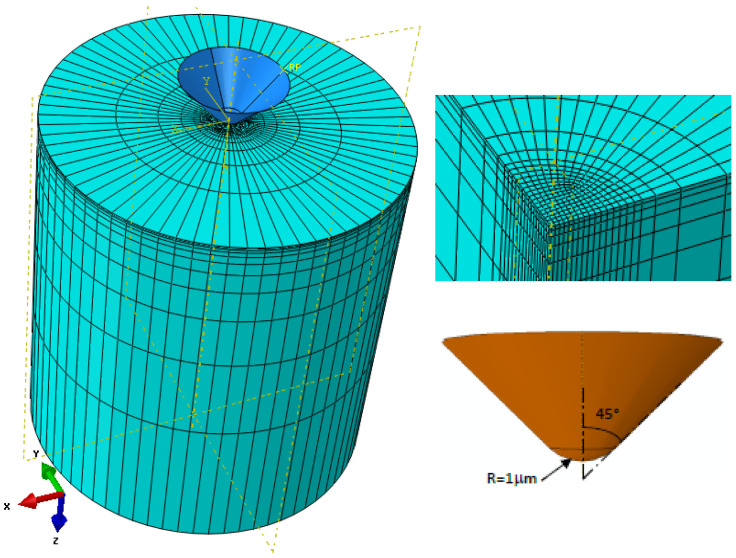
Finite element mesh and indenter geometry.

The selection of the mesh size is generally a compromise between the computational cost and the solution accuracy. However, to describe the deformation and stress gradients as well as the contact area between the indenter and specimen with sufficient accuracy, the mesh near the indenter needs to be finer. After a mesh sensitivity analysis, the sample was discretized using 13,860 eight-node linear hexahedral elements with a reduced integration scheme (C3D8R), and the smallest element size was 60 nm. To keep the simulations computationally tractable, the mesh density was progressively coarsened away from the contact zone (Figure 3).

Furthermore, based on experimental conditions, all simulations were performed under quasi-static, isothermal load-controlled conditions. In addition, the nonlinearity option was used to account for both material and geometric (finite strains and rotations) nonlinearities.

### 3.3. Inverse Identification Procedure of Crystal Plasticity Model Parameters

An inverse optimization strategy was developed to determine the crystal plasticity model parameters using both the load-penetration depth curves from nanoindentation experiments and profiles of residual indentation (penetration depth profile after unloading) from AFM measurements. The adopted inverse optimization strategy consisted of coupling the finite element simulation of the nanoindentation experiment and the advanced optimization algorithm MOGA-II proposed by Poloni et al. [62]. It uses a smart multi-search elitism for robustness and directional crossover for fast convergence. Its efficiency is ruled by its operators (classical crossover, directional crossover, mutation, and selection) and by using elitism. Compared with other algorithms, the dominant characteristics of MOGA-II are robust stability, low susceptibility for ending up in a local optimum, and adaptability for nonlinear problems [63]. The set of initial designs is generated by means of the pseudo-random Sobol sequence [64].

The used objective function is defined by a least-squares scalar function of the difference between the numerical calculations and experimental measurements of the load-penetration depth curve and the deviation of the maximum pile-up height, given by:

(6) $$F_{obj}\left(x\right)=\frac{1}{N}\overset{N}{\underset{i=1}{\sum }}\left(\beta_{1}{\left(h_{FEM}\left(x,t_{i}\right)-h_{exp}\left(t_{i}\right)\right)}^{2}+\beta_{2}{\left(pl_{FEM}\left(x\right)-pl_{exp}\right)}^{2}\right)$$

where x is the vector of unknown parameters, N is the number of data set, $t_{i}$ is the time of the corresponding experimental point i, $h_{FEM}\left(x,t_{i}\right)$ and $h_{exp}\left(t_{i}\right)$ are the indenter displacements computed by FEM and measured experimentally, respectively, $pl_{FEM}\left(x\right)$ and $pl_{exp}$ are the maximum pile-up heights computed by FEM and measured experimentally on the surface of residual imprint, respectively, and $\beta_{1}$ and $\beta_{2}$ are weighting coefficients.

**Figure 4:**
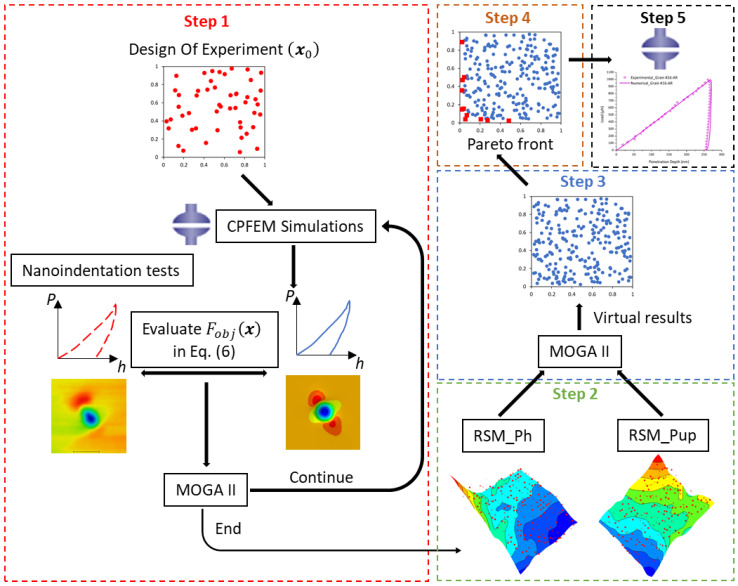
Flow chart of the proposed optimization procedure.

The optimization process to determine the crystal plasticity model parameters was carried out according to the flow chart in Figure 4, with the following steps.

- Step 1: Develop an optimization strategy by coupling between a finite element analysis and optimization algorithm.
  1. Select and extract the experimental indentation data of the load-penetration depth curve and the penetration depth profile after unloading.
  2. Run the CPFEM simulation using ABAQUS software.
  3. Extract the load-penetration depth and penetration depth profile after unloading from the CPFEM results using Python script.
  4. Evaluate the objective function given by Equation (6).
  5. Update the set of crystal plasticity model parameters using the MOGA-II genetic algorithm.
  6. Repeat steps 2–5 until a large enough population is obtained from the MOGA-II genetic algorithm.

Practically, each ABAQUS simulation took approximately 120 min on a Dell Precision T3610 Intel Xeon CPU E5-1620 v2 (3.70 GHz). The initial population was a set of 8 designs, and the number of MOGA-II generations was set to 12.

- Step 2: Build surrogate models.

The population of the successfully converged values in the design table of the first step is used to build surrogate models (or metamodels), applying the response surface methodology (RSM) [65]. It consists in building approximation functions to represent the response functions which are unknown. The Gaussian Processes technique [66] is used to build the approximation functions for two responses: the load-penetration depth (RSM_Ph) and the pile-up height (RSM_Pup), respectively.

- Step 3: Optimization process using metamodels.

The metamodels obtained in Step 2 are used to perform a multi-objective optimization process using the MOGA-II algorithm. The DOE table is constructed by selecting the 12 best designs from the results of the first optimization process, and the number of generations is set to 200. Since the metamodels are analytical, the simulations took a few minutes. This process creates a set of virtual values.

- Step 4: Selection of the best designs.

From the virtual design values, we choose the set of result points constituted by Pareto front, giving a set of optimal solutions.

- Step 5: Find the optimal solution.

The Pareto set is finally used to run CPFEM simulations to obtain the real values and then the best one is considered as the optimal solution.

## 4. Results

In the following sections, the identification of the crystal plasticity model parameters (n, ${\dot{\gamma }}_{0}^{\alpha }$, $h_{0}^{\alpha }$, $\tau_{0}^{\alpha }$, $\tau_{s}^{\alpha }$) was carried out with the method described in the previous section using the load vs. penetration curve and residual imprint of Grain #10. Grains #11, #16, and #18 at the same load level and Grain #18 at a higher load level were then used for the validation.

### 4.1. Results of Inverse Identification of Model Parameters

Since the basal glide is the easy slip system for zinc, it is taken as the reference system. We also assumed constant ratios of the initial resolved shear stresses between basal and prismatic and pyramidal systems. From the literature review, we have chosen the ratios: $\tau_{prism}/\tau_{basal}=15$ and $\tau_{py\langle c+a\rangle }/\tau_{basal}=10$ [10,14,26].

To keep the inverse identification procedure more tractable, we also assumed the same reference shear strain rate for all slip systems (${\dot{\gamma }}_{0}^{\alpha }={\dot{\gamma }}_{0}=0.01\;s^{-1}$). In nanoindentation tests, the response of the material is affected by the loading strain rate. Xiao et al. [67] showed that the slopes of contact stiffness–depth curve, modulus, and hardness of cured isotropic conductive adhesive increase with the increasing loading strain rate. Mao et al. [68] showed that the hardness and creep flow of the tripler plane of potassium dihydrogen phosphate crystal increase with the loading strain rate. Znemah et al. [69] analyzed the effects of strain rate and testing temperature on the hardness of Inconel 718 manufactured by selective laser melting (SLM) and measured by nanoindentation. In our study, all the nanoindentation tests were conducted at the same strain rate and the effect of the strain rate was not studied. Therefore, we assume a constant strain rate sensitivity exponent (n=25) according to Cauvin et al. [12]. Finally, the number of crystal plasticity model parameters to identify was reduced to the following seven parameters: $h_{0}^{basal}$, $h_{0}^{prism}$, $h_{0}^{py\langle c+a\rangle }$, $\tau_{0}^{basal}$, $\tau_{s}^{basal}$, $\tau_{s}^{prism}$, and $\tau_{s}^{py\langle c+a\rangle }$.

**Figure 5:**
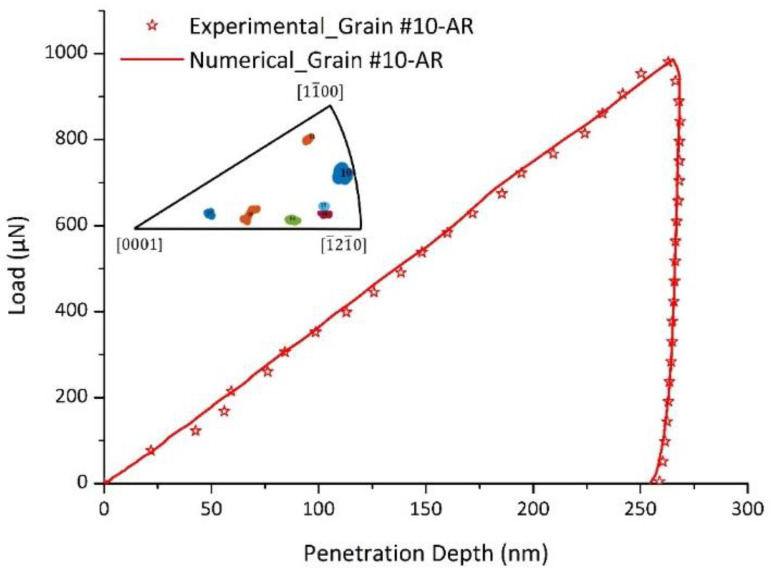
Experimental and numerical load-penetration depth curves for Grain #10.

**Figure 6:**
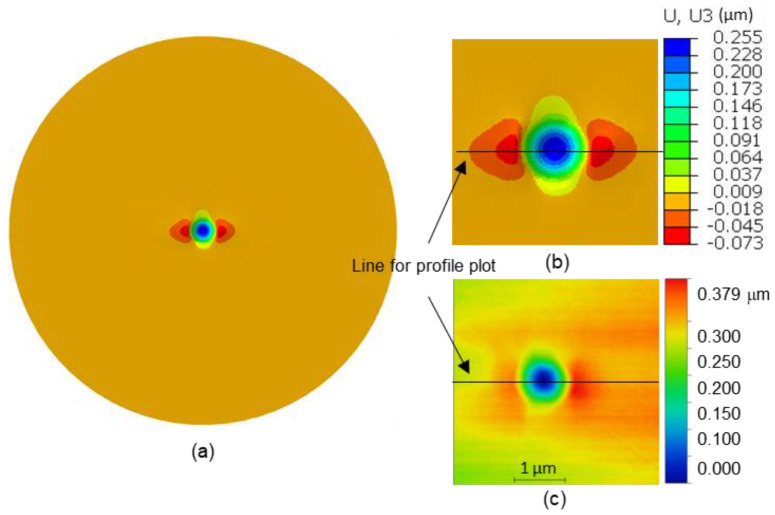
Out-of-plane displacements on the top surface of Grain #10 after unloading: (**a**,**b**) simulation results, (**c**) AFM image.

**Figure 7:**
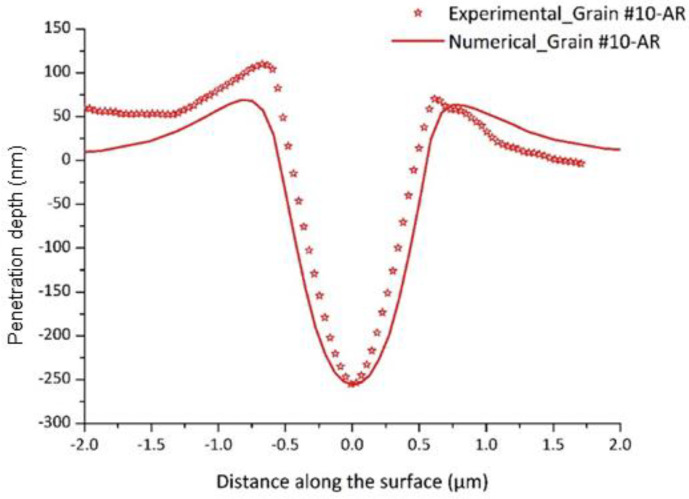
Experimental and numerical penetration depth profile after unloading for Grain #10.

The identified parameters are presented in Table 5. Figure 5 shows the experimental load-penetration depth curve and the numerical one issued from the identification procedure. One can notice the very good agreement between the experimental data and the simulated response of Grain #10. The distribution of simulated out-of-plane displacements on the top sample surface and AFM image corresponding to the imprint left by the indenter after unloading for Grain #10 are depicted in Figure 6, showing an overall similarity with an unsymmetrical two-fold pile-up in both experimental and numerical results. Experimental and numerical profiles along a line crossing the pile-up maxima are extracted and plotted in Figure 7.

### 4.2. Validation of the Identified Model on Different Grain Orientations

**Figure 8:**
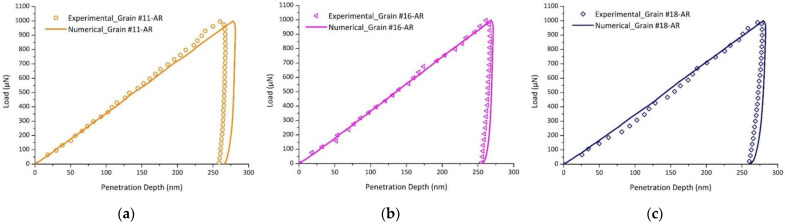
Experimental and numerical load-penetration depth curves for: (**a**) Grain #11, (**b**) Grain #16, and (**c**) Grain #18.

**Figure 9:**
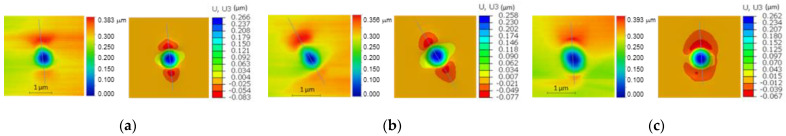
AFM images and CPFEM imprint after unloading: (**a**) Grain #11, (**b**) Grain #16, and (**c**) Grain #18.

**Figure 10:**
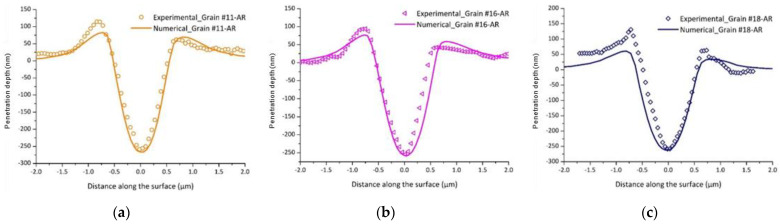
Experimental and numerical penetration depth profiles after unloading for: (**a**) Grain #11, (**b**) Grain #16, and (**c**) Grain #18.

To verify the validity of the proposed model, indentation tests performed on grains of different crystalline orientations (Grains #11, #16, and #18) were simulated using the CPFEM model using the identified parameters presented in Table 5. Figure 8 shows a comparison of the experimental and numerical load-penetration depth curves for Grains #11, #16, and #18. For all grains, a good agreement between the experimental data and the simulated responses was observed. The AFM image and CPFEM imprint left by the indenter after unloading for different grain orientations and the penetration depth profiles for Grains #11, #16, and #18 are shown in Figure 9 and Figure 10, respectively. One can notice that the penetration depth profiles after unloading were accurately captured by the CPFEM model. It is important to point out that these grains have different crystallographic orientations than Grain #10 but the same maximum load level of 1000 µN, and were not used in the identification process.

### 4.3. Validation of the Identified Model with a Higher Load Level

A nanoindentation test performed on Grain #18 at a higher load level (i.e., F = 5000 μN) was simulated using the identified CPFEM model. The experimental test was carried out far from the initial indentation area.

To minimize the boundary effects in the finite element simulation, we have chosen a sample with 50 μm diameter and 50 μm height for the case of a higher load level. This size assures that the maximum contact radius always remains smaller than R/50, where R is the radial dimension of the cylindrical sample [70]. We also used a refined mesh in the contact zone, consisting of 22,680 eight-node linear hexahedral elements with a reduced integration scheme, C3D8R. The smallest element size equals to 60 nm, as in the case of the identified model.

**Figure 11:**
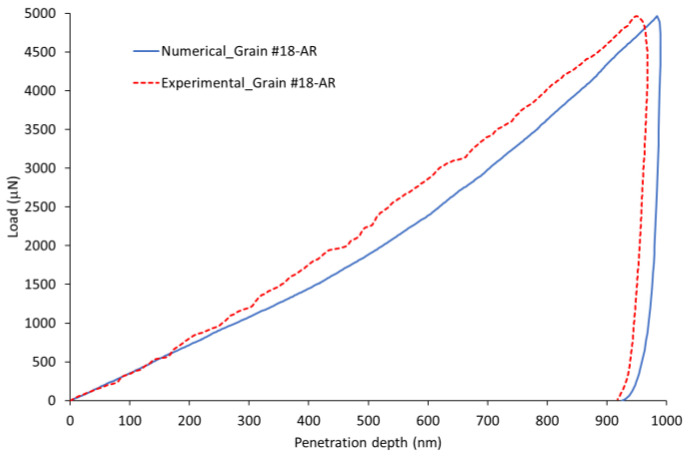
Experimental and numerical load vs. penetration depth curves for Grain #18 at a load level of F = 5000 μN.

**Figure 12:**
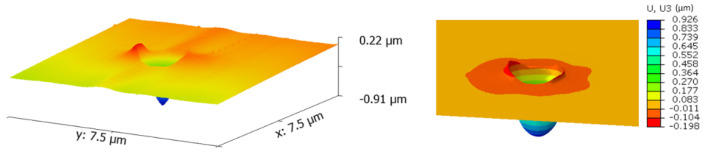
Comparison of 3D AFM and simulation out-of-plane displacements on the top surface of Grain #18 after unloading at a load level of F = 5000 μN.

**Figure 13:**
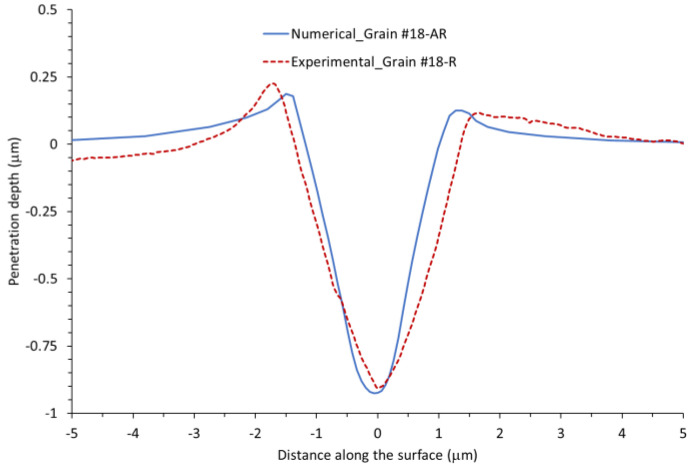
Experimental and numerical penetration depth profile after unloading for Grain #18 at a load level of F = 5000 μN.

A comparison of experimental and numerical nanoindentation results for Grain #18 with applied load F = 5000 μN is summarized in Figure 11, Figure 12 and Figure 13. An overall similarity was found between experimental and CPFEM results for the load vs. penetration depth curve (Figure 11), residual indentation imprint (Figure 12), and penetration depth profile after unloading (Figure 13). The model overestimates the penetration depth after unloading by 2.25% and underestimates the maximum pile-up by about 14%.

## 5. Discussion

### 5.1. Identifiability Results

The identification of parameters for CPFEM from datasets can be especially complicated due to the multitude of deformation modes, size of the datasets, and the approximation of the material physics. Thus, a computationally efficient and robust approach to the identification of CPFEM parameters is sought. The identification of the parameters of the CPFEM using indentation curves and residual topographies has been evaluated. Indeed, the imprint mapping is a richer observation than the indentation curve, as concluded by Bolzon et al. [71] and Renner et al. [72]. We have also leveraged an expensive function optimization strategy of constructing a surrogate model. By searching a response surface using the MOGA-II genetic algorithm (GA) and selectively updating the response surface using the CPFEM, a robust set of parameters was identified from indentation curves and residual topographies with limited CPFEM calls. This approach, using the response surface methodology together with a genetic algorithm to determine an optimal set of parameters, was introduced by Sedighiani et al. [73] and extended by Savage et al. [74] by using the multi-objective GA (MGA) formalism to investigate the Pareto optimal set of solutions.

### 5.2. Crystal Resolved Shear Stress (CRSS)

The CPFEM has provided a good agreement with the experiment in terms of load vs. penetration depth curves (Figure 5, Figure 8 and Figure 11), residual indentation imprint (Figure 6, Figure 9 and Figure 12), and penetration depth profile after unloading (Figure 7, Figure 10 and Figure 13). The parameter identification procedure proposed in this study resulted in the parameters provided in Table 5 that have satisfied different CPFEM simulations of nanoindentations on different crystal orientations.

From the parameters in Table 5, the basal slip system has a CRSS of about 2 MPa. For pure zinc, the CRSS for the basal slip system ranges from 0.14 to 5 MPa, and thus lies within a reasonable range, based on the literature as summarized in Table 1.

### 5.3. Grain Slip Activities

**Figure 14:**
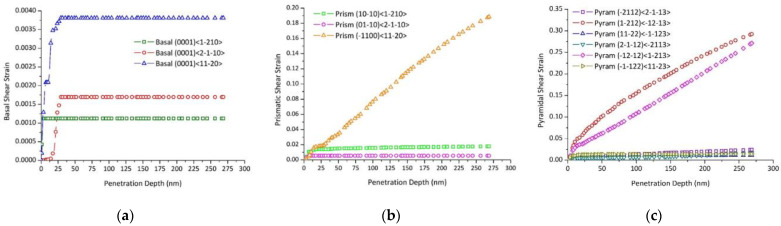
Grain #10 slip activities: (**a**) basal system, (**b**) prismatic system, and (**c**) pyramidal <c + a> system.

An analysis of slip system activities in a point located underneath the indenter is presented in Figure 14 for Grain #10, where the evolution of shear strain on all slip systems is plotted. We can notice that small shear strains were achieved with basal glide and the three systems were activated. Higher shear strains were achieved with prismatic glide (≈0.2) and the three systems were activated. The highest shear strains were achieved with the second-order pyramidal glide (≈0.3) and the six systems were activated. Since the basal slip is the easiest deformation mode in zinc, basal systems $\left\{0001\right\}\langle 1\bar{2}10\rangle$, $\left\{0001\right\}\langle 2\bar{11}0\rangle$, and $\left\{0001\right\}\langle 11\bar{2}0\rangle$ were activated early, but they saturated at a low penetration depth (<25 nm). Prismatic systems $\left\{10\bar{1}0\right\}\langle 1\bar{2}10\rangle$ and $\left\{01\bar{1}0\right\}\langle 2\bar{11}0\rangle$ were activated early and saturated at an early deformation stage (h ≈ 10 nm). However, the prismatic system $\left\{\bar{1}100\right\}\langle 11\bar{2}0\rangle$ was the most active prismatic system and the saturation was not observed in the penetration depth range of 0 to 275 nm. The activities of pyramidal systems $\left\{\bar{2}112\right\}\langle 2\bar{11}3\rangle$, $\left\{11\bar{2}2\right\}\langle \bar{11}23\rangle$, $\left\{2\bar{11}2\right\}\langle \bar{2}113\rangle$, and $\left\{\bar{11}22\right\}\langle 11\bar{2}3\rangle$ were very low for Grain #10, while pyramidal systems $\left\{1\bar{2}12\right\}\langle \bar{1}2\bar{1}3\rangle$ and $\left\{\bar{1}2\bar{1}2\right\}\langle 1\bar{2}13\rangle$ were the most active systems and did not saturate in the penetration depth range of 0 to 275 nm.

### 5.4. Orientation-Dependent Indent Topography

To analyze indent topography, three different orientations were considered: basal (Grain #18), prismatic (Grain #11), and pyramidal (Grain #16). We can notice in Figure 9 and Figure 10 that pile-up sizes and distributions showed significant asymmetries. The results in all three cases revealed pile-up rather than sink-in behavior. The piling up occurs only in limited zones around the indents, that is the pile-up patterns are strictly crystallographic and orientation dependent [12]. The appearance of such pile-up patterns is generally interpreted in terms of the stress-hardening behavior of the indented material [47,75,76]. According to these works, the surface underneath the indent tends to pile-up against the indenter when the indented specimen is highly pre-strained with only little reserves for further work-hardening, or has generally a low strain-hardening potential.

**Figure 15:**
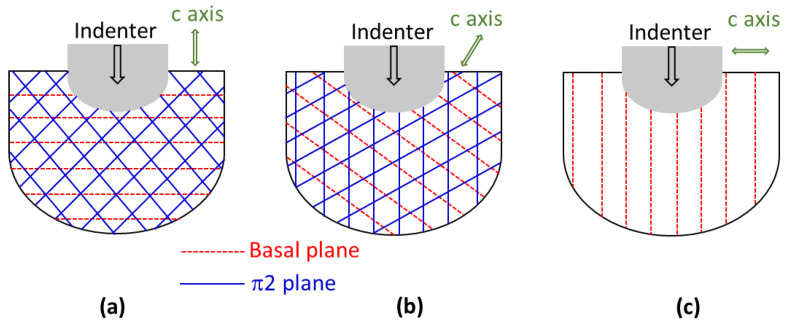
Schematics showing activated slip systems for perfect orientations: (**a**) basal, (**b**) pyramidal, and (**c**) prismatic.

The perfect basal and second-order pyramidal planes’ orientations are schematically shown in Figure 15a with respect to the nanoindentation direction, which is parallel to the c-axis. It is clear in this case that the second-order pyramidal slip system should activate as indentation is parallel to the c-axis 〈0001〉. The basal slip system could also activate. Kwon et al. [77] and Sarvesha et al. [78] have observed second-order slip lines during indentation parallel to the c-axis 〈0001〉 for Zn and α-Ti alloys, respectively. Since basal planes are parallel to the surface of the crystal, Kwon et al. [77] used site-specific cross-section TEM to observe basal slip lines below the indenter.

## 6. Conclusions

The work carried out in this paper concerns the characterization and modeling of the mechanical behavior of a high-purity polycrystalline zinc using nanoindentation tests and CPFEM. We carried out nanoindentation tests with a sphero-conical tip on grains of different crystallographic orientations. These tests led to load-penetration depth curves and residual imprint images, acquired by atomic force microscopy (AFM) in contact mode, allowing to measure penetration depth profiles after unloading. We considered that each grain of a given crystallographic orientation behaved like a single crystal, and that the dominant mechanism of deformation is the crystallographic slip according to the basal, prismatic, and second-order pyramidal systems. We developed an approach to identify the laws of crystalline plasticity based on the exploitation of nanoindentation measurements and the finite element calibration. An inverse method has been used, which consists of coupling 3D numerical simulation by the finite element method of the nanoindentation test with the optimization of the constitutive law parameters by means of a genetic algorithm using response surface methodology (RSM). The results obtained by identification are in good agreement with the experimental results for the load-penetration depth curves, for the morphology and location of the pile-ups, and for the penetration depth profiles after unloading. The proposed optimization algorithm can be readily applied for other HCP materials. Optimization results can be improved by using nanoindentation tests with other indenters (Berkovich and Cube corner). On the other hand, performing tests with different shear rates could help to identify the strain rate sensitivity exponent.

## Figures and Tables

**Table 1:** Values of the critical resolved shear stress for zinc reported in the literature.

Material	$\tau_{basal}$ (MPa)	$\tau_{prism}$ (MPa)	$\tau_{\pi 2}$ (MPa)	$\tau_{twin}$ (MPa)	$\tau_{prism}/\tau_{basal}$	$\tau_{\pi 1}/\tau_{basal}$	$\tau_{\pi 2}/\tau_{basal}$	$\tau_{twin}/\tau_{basal}$	Ref.
Pure zinc	0.28								[22]
Pure zinc	0.3		10–15						[8]
Pure zinc	0.14–0.28		0.7–1.9				30		[32]
Pure zinc	0.7								[13]
Pure zinc	0.5–6								[33]
Pure zinc	1	10	5						[34]
Pure zinc	0.4	6–15	4–10						[35]
Pure zinc	1.5	22.5	15						[36]
Pure zinc	5		20						[11]
Zn-0.16%Cu-0.076%Ti					15		10	30	[10]
Zn-0.165%Cu-0.07%Ti					15	30	10		[25]
Zn-0.17%Cu-0.08%Ti					15		2	30	[26]
Zn-0.1%Ti	5–12.5								[20]
Zn-0.2%Al					15	10	10	8	[29]
Zn-0.001%Fe-0.2%Al	1.5	22.5	15	25					[27]
Zn-0.08%Cu-0.1%Ti	7	180	75	200					[30]
Zn-(0.1–0.2%)Cu-Ti	1		47.94	106.25					[12]

**Table 2:** Chemical composition (in ppm) of AR high-purity (99.99%) polycrystalline zinc.

Ag	Cd	Ca	Cr	Cu	Cs	Fe	Mn	Ni	Pd	Pb	Rh	Ta	Tl	Ti
0.36	3.4	<0.1	0.55	4	<0.1	4.9	0.18	0.66	<1	12	<0.5	<10	5.1	4.5

**Table 3:** Crystalline orientation of the selected grains.

Grain #	Crystalline Orientation	Bunge Angles (°)		
		$\varphi_{1}$	ϕ	$\varphi_{2}$
10	Pyramidal	105.81	94.40	192.38
11	Prismatic	3.89	107.01	214.74
16	Pyramidal	158.75	72.58	184.56
18	Basal	0.04	137.10	233.02

**Table 4:** Elastic constants of zinc single crystal [57].

$C_{11}\;(\mathrm{GPa})$	$C_{33}\;(\mathrm{GPa})$	$C_{44}\;(\mathrm{GPa})$	$C_{12}\;(\mathrm{GPa})$	$C_{13}\;(\mathrm{GPa})$
165	61.8	39.6	31.1	50

**Table 5:** Identified crystal plasticity model parameters for zinc single crystal.

$h_{0}^{basal}$ (MPa)	$h_{0}^{prism}$ (MPa)	$h_{0}^{py\langle c+a\rangle }$ (MPa)	$\tau_{0}^{basal}$ (MPa)	$\tau_{s}^{basal}$ (MPa)	$\tau_{s}^{prism}$ (MPa)	$\tau_{s}^{py\langle c+a\rangle }$ (MPa)
107	188	85.9	2.01	18.5	104	42

## Data Availability

The data are not publicly available due to privacy restrictions.

## References

[1] Marder A.R. (2000). The metallurgy of zinc-coated steel. Prog. Mater. Sci..

[2] Diler E., Rouvellou B., Rioual S., Lescop B., Nguyen Vien G., Thierry D. (2014). Characterization of corrosion products of Zn and Zn Mg–Al coated steel in a marine atmosphere. Corros. Sci..

[3] Li J., Wang Q., Gao N., Nwokolo I.K., Zhang W., Ma L., Liu F., Han E.-H. (2021). Interface Characteristics and Anticorrosion Performances of Cold Galvanizing Coatings Incorporated with γ-chloropropyl Triethoxysilane on Hot-Dip Galvanized Steel. Coatings.

[4] Liu Y.W., Wang Z.Y., Cao G.W., Cao Y., Huo Y. (2017). Study on corrosion behavior of zinc exposed in coastal-industrial atmospheric environment. Mater. Chem. Phys..

[5] Yuan Y., Liu X., Pu G., Wang T., Guo Q. (2021). Corrosion features and time-dependent corrosion model of Galfan coating of high strength steel wires. Constr. Build. Mater..

[6] Khezrloo A., Rezazadeh F., Rajaee M., Tayebi M., Aghaie E., Behnamian Y. (2021). Effect of coating parameters on microstructure, corrosion behavior, hardness and formability of hot-dip Galfan and galvanized coatings. Int. J. Mater. Res..

[7] Zhang X., Odnevall Wallinder I., Leygraf C. (2018). Atmospheric corrosion of Zn–Al coatings in a simulated automotive environment. Surf. Eng..

[8] Bell R.L., Cahn R.W. (1957). The Dynamics of Twinning and the Interrelation of Slip and Twinning in Zinc Crystals. Proc. R. Soc. A Math. Phys. Sci..

[9] Antonopoulos J.G., Karakostas T., Komninou P., Delavignette P. (1988). Dislocation Movements and Deformation Twinning in Zinc. Acta Metall..

[10] Philippe M.J., Wagner F., Mellab F.E., Esling C., Wegria J. (1994). Modelling of texture evolution for materials of hexagonal symmetry—I. Application to zinc alloys. Acta Metall. Et Mater..

[11] Hagihara K., Mayama T., Honnami M., Yamasaki M., Izuno H., Okamoto T., Ohashi T., Nakano T., Kawamura Y. (2016). Orientation dependence of the deformation kink band formation behavior in Zn single crystal. Int. J. Plast..

[12] Cauvin L., Raghavan B., Bouvier S., Wang X., Meraghni F. (2018). Multi-scale investigation of highly anisotropic zinc alloys using crystal plasticity and inverse analysis. Mater. Sci. Eng. A.

[13] Barendreght J.A., Sharpe W.N. (1973). The effect of biaxial loading on the critical resolved shear stress of zinc single crystals. J. Mech. Phys. Solids.

[14] Fundenberger J.J., Philippe M.J., Wagner F., Esling C. (1997). Modelling and prediction of mechanical properties for materials with hexagonal symmetry (zinc, titanium and zirconium alloys). Acta Mater..

[15] Turner P.A., Tomé C.N. (1994). A study of residual stresses in Zircaloy-2 with rod texture. Acta Metall. Mater..

[16] Lebensohn R.A., Tomé C.N. (1993). A self-consistent anisotropic approach for the simulation of plastic deformation and texture development of polycrystals: Application to zirconium alloys. Acta Metall. Et Mater..

[17] Britton T.B., Liang H., Dunne F.P.E., Wilkinson A.J. (2010). The effect of crystal orientation on the indentation response of commercially pure titanium: Experiments and simulations. Proc. R. Soc. A Math. Phys. Eng. Sci..

[18] Zambaldi C., Yang Y., Bieler T., Raabe D. (2012). Orientation informed nanoindentation of α-titanium: Indentation pileup in hexagonal metals deforming by prismatic slip. J. Mater. Res..

[19] Mikułowski B., Boczkal G. (2009). Zn-Ti Single Crystals Deformed along the Basal Slip System. Arch. Metall. Mater..

[20] Boczkal G., Mikułowski B. (2004). Precipitation hardening of Zn0.1 at.%Ti single crystals deformed on the (0001)<11–20> system. J. Alloys Compd..

[21] Harper S., Cottrell A.H. (1950). Surface Effects and the Plasticity of Zinc Crystals. Proc. Phys. Soc. Sect. B.

[22] Edwards E.H., Washburn J. (1954). Strain Hardening of Latent Slip Systems in Zinc Crystals. JOM.

[23] Adams K.H., Vreeland T., Wood D.S. (1967). Basal Dislocation Mobility in Zinc Single Crystals. Mater. Sci. Eng..

[24] Rogers D.H., Roberts W.T. (1968). Plastic Anisotropy of Titanium and Zinc Sheet—II. Int. J. Mech. Sci..

[25] Diot M., Fundenberger J.J., Philippe M.J., Esling C., Wegria J. (1998). Texture gradient in rolled zinc sheets. Scr. Mater..

[26] Zhang F., Vincent G., Sha Y.H., Zuo L., Fundenberger J.J., Esling C. (2004). Experimental and Simulation Textures in an Asymmetrically Rolled Zinc Alloy Sheet. Scr. Mater..

[27] Parisot R., Forest S., Gourgues A.F., Pineau A., Mareuse D. (2000). Modeling the Mechanical Behavior of a Multicrystalline Zinc Coating on a Hot-Dip Galvanized Steel Sheet. Comput. Mater. Sci..

[28] Parisot R., Forest S., Pineau A., Grillon F., Demonet X., Mataigne J.M. (2004). Deformation and Damage Mechanisms of Zinc Coatings on Hot-Dip Galvanized Steel Sheets: Part I. Deformation Modes. Metall. Mater. Trans. A.

[29] Vincent G., Zhang F., Fundenberger J.J., Esling C. (2005). Experimental and Simulation Textures in an Asymmetrically Skin-Passed Zinc Galvanized Sheet. Scr. Mater..

[30] Borodachenkova M., Wen W., Barlat F., Pereira A., Grácio J. (2015). Modeling of the Mechanical Behavior and Texture Evolution in Zn Alloys during Reverse Shear Loading. J. Mater. Process. Technol..

[31] Peirce D., Asaro R.J., Needleman A. (1983). Material Rate Dependence and Localized Deformation in Crystalline Solids. Acta Metall..

[32] Stofel E.J. (1962). Plastic Flow and Fracture of Zinc Single Crystals. Ph.D. Thesis.

[33] Bosin M.E., Lavrentev F.F., Nikiforenko V.N. (1998). Nonlinearity and Localization of Plastic Deformation in Zn Single Crystals with Forest Dislocation. Mater. Sci. Eng. A.

[34] Solas D.E., Tome C.N., Engler O., Wenk H.R. (2001). Deformation and Recrystallization of Hexagonal Metals: Modeling and Experimental Results for Zinc. Acta Mater..

[35] Lassila D.H., Leblanc M.M., Florando J.N. (2007). Zinc Single Crystal Deformation Experiments Using a 6 Degrees of Freedom Apparatus. Metall. Mater. Trans. A.

[36] Casals O., Forest S. (2009). Finite Element Crystal Plasticity Analysis of Spherical Indentation in Bulk Single Crystals and Coatings. Comput. Mater. Sci..

[37] Zhu W., Hughes J.J., Bicanic N., Pearce C.J. (2007). Nanoindentation Mapping of Mechanical Properties of Cement Paste and Natural Rocks. Mater. Charact..

[38] Hu C., Li Z. (2015). A Review on the Mechanical Properties of Cement-Based Materials Measured by Nanoindentation. Constr. Build. Mater..

[39] Abbès F., Abbès B., Benkbou R., Asroun A. (2020). A FEM Multiscale Homogenization Procedure using Nanoindentation for High Performance Concrete. J. Appl. Comput. Mech..

[40] Li H., Vlassak J.J. (2009). Determining the Elastic Modulus and Hardness of an Ultra-Thin Film on a Substrate Using Nanoindentation. J. Mater. Res..

[41] Esqué-de los Ojos D., Očenášek J., Alcalá J. (2014). Sharp indentation crystal plasticity finite element simulations: Assessment of crystallographic anisotropy effects on the mechanical response of thin FCC single crystalline films. Comput. Mater. Sci..

[42] Jian S.R., Lee Y.H. (2014). Nanoindentation-induced interfacial fracture of ZnO thin films deposited on Si (1 1 1) substrates by atomic layer deposition. J. Alloys Compd..

[43] Li X., Bhushan B., Takashima K., Baek C.W., Kim Y.K. (2003). Mechanical characterization of micro/nanoscale structures for MEMS/NEMS applications using nanoindentation techniques. Ultramicroscopy.

[44] Kim J.H., Yeon S.C., Jeon Y.K., Kim J.G., Kim Y.H. (2003). Nano-indentation method for the measurement of the Poisson’s ratio of MEMS thin films. Sens. Actuators A Phys..

[45] Palacio M.L., Bhushan B. (2013). Depth-sensing indentation of nanomaterials and nanostructures. Mater. Charact..

[46] Chiu Y.L., Ngan A.H.W. (2002). Time-dependent characteristics of incipient plasticity in nanoindentation of a Ni3Al single crystal. Acta Mater..

[47] Wang Y., Raabe D., Klüber C., Roters F. (2004). Orientation dependence of nanoindentation pile-up patterns and of nanoindentation microtextures in copper single crystals. Acta Mater..

[48] Liu M., Lin J.Y., Lu C., Tieu K.A., Zhou K., Koseki T. (2017). Progress in indentation study of materials via both experimental and numerical methods. Crystals.

[49] Oliver W.C., Pharr G.M. (1992). An Improved Technique for Determining Hardness and Elastic Modulus Using Load and Displacement Sensing Indentation Experiments. J. Mater. Res..

[50] Campbell J.E., Thompson R.P., Dean J., Clyne T.W. (2018). Experimental and computational issues for automated extraction of plasticity parameters from spherical indentation. Mech. Mater..

[51] Li Y.G., Kanouté P., François M., Chen D., Wang H.W. (2019). Inverse identification of constitutive parameters with instrumented indentation test considering the normalized loading and unloading P-h curves. Int. J. Solids Struct..

[52] Pöhl F. (2019). Determination of unique plastic properties from sharp indentation. Int. J. Solids Struct..

[53] Long X., Jia Q.P., Li Z., Wen S.X. (2020). Reverse analysis of constitutive properties of sintered silver particles from nanoindentations. Int. J. Solids Struct..

[54] Long X., Jia Q.P., Shen Z., Liu M., Guan C. (2021). Strain rate shift for constitutive behaviour of sintered silver nanoparticles under nanoindentation. Mech. Mater..

[55] Nguyen N.P.T., Abbès F., Abbès B., Li Y. (2017). Orientation-dependent response of pure zinc grains under instrumented indentation: Micromechanical modeling. International Conference on Advances in Computational Mechanics.

[56] Nguyen N.P.T., Abbès F., Abbès B., Lecomte J.-S., Schuman C. (2019). Experimental Characterisation and Numerical Modelling of the Effect of Cold Rolling on the Nanoindentation Response of Pure Zinc Grains. IOP Conf. Ser. Mater. Sci. Eng..

[57] Tromans D. (2011). Elastic Anisotropy of HCP Metal Crystals and Polycrystals. Int. J. Res. Rev. Appl. Sci..

[58] Asaro R.J., Needleman A. (1985). Texture Development and Strain Hardening in Rate Dependent Polycrystals. Acta Metall..

[59] Marin E.B., Dawson P.R. (1998). Elastoplastic Finite Element Analyses of Metal Deformations Using Polycrystal Constitutive Models. Comput. Methods Appl. Mech. Eng..

[60] Zhao M., Ogasawara N., Chiba N., Chen X. (2006). A new approach to measure the elastic–plastic properties of bulk materials using spherical indentation. Acta Mater..

[61] Bhattacharya A.K., Nix W.D. (1988). Finite Element Simulation of Indentation Experiments. Int. J. Solids Struct..

[62] Poloni C., Pediroda V., Quagliarella D., Périaux J., Poloni C., Winter G. (1997). GA coupled with computationally expensive simulations: Tools to improve efficiency. Genetic Algorithms and Evolution Strategies in Engineering and Computer Science.

[63] Bandyopadhyay S., Saha S. (2013). Use of Multiobjective Optimization for Data Clustering. Unsupervised Classification.

[64] Sobol I.M. (1967). On the distribution of points in a cube and the approximate evaluation of integrals. USSR Comput. Math. Math. Phys..

[65] Myers R.H., Montgomery D.C., Anderson-Cook C.M. (2009). Response Surface Methodology: Process and Product Optimization Using Designed Experiments.

[66] MacKay D.J. (1998). Introduction to Gaussian processes. NATO ASI Ser. F Comput. Syst. Sci..

[67] Xiao G., Li Z., Liu E., Qiao L., Shu X., Sun R. (2021). Microscale mechanical properties dependent on the strain rate and temperature of cured isotropic conductive adhesive. Mech. Time-Depend. Mater..

[68] Mao J., Liu W., Li D., Zhang C., Ma Y. (2021). The Strain Rate Sensitivity and Creep Behavior for the Tripler Plane of Potassium Dihydrogen Phosphate Crystal by Nanoindentation. Micromachines.

[69] Abo Znemah R., Voyiadjis G.Z., Wood P., Akbari E. (2022). Strain Rate and Temperature Effects in Nanoindentation Testing on Hardness in Selective Laser Melting IN718. ASME J. Eng. Mater. Technol..

[70] Giannakopoulos A.E., Larsson P.L., Vestergaard R. (1994). Analysis of Vickers Indentation. Int. J. Solids Struct..

[71] Bolzon G., Maier G., Panico M. (2004). Material model calibration by indentation, imprint mapping and inverse analysis. Int. J. Solids Struct..

[72] Renner E., Bourceret A., Gaillard Y., Amiot F., Delobelle P., Richard F. (2020). Identifiability of single crystal plasticity parameters from residual topographies in Berkovich nanoindentation on FCC nickel. J. Mech. Phys. Solids.

[73] Sedighiani K., Diehl M., Traka K., Roters F., Sietsma J., Raabe D. (2020). An efficient and robust approach to determine material parameters of crystal plasticity constitutive laws from macro-scale stress–strain curves. Int. J. Plast..

[74] Savage D.J., Feng X., Knezevic M. (2021). Identification of crystal plasticity model parameters by multi-objective optimization integrating microstructural evolution and mechanical data. Comput. Methods Appl. Mech. Eng..

[75] Nix W.D. (1997). Elastic and plastic properties of thin films on substrates: Nanoindentation techniques. Mater. Sci. Eng. A.

[76] Chaudhri M.M., Winter M. (1988). The load-bearing area of a hardness indentation. J. Phys. D Appl. Phys..

[77] Kwon J., Brandes M.C., Sudharshan Phani P., Pilchak A.P., Gao Y.F., George E.P., Pharr G.M., Mills M.J. (2013). Characterization of deformation anisotropies in an α-Ti alloy by nanoindentation and electron microscopy. Acta Mater..

[78] Sarvesha R., Gokhale A., Kumar K., Sharma N.K., Jain J., Singh S.S. (2020). Effect of crystal orientation on indentation-induced deformation behavior of zinc. Mater. Sci. Eng. A.

